# Why some women fail to give birth at health facilities: A comparative study between Ethiopia and Nigeria

**DOI:** 10.1371/journal.pone.0196896

**Published:** 2018-05-03

**Authors:** Sanni Yaya, Ghose Bishwajit, Olalekan A. Uthman, Agbessi Amouzou

**Affiliations:** 1 School of International Development and Global Studies, University of Ottawa, Ottawa, ON, Canada; 2 Warwick Centre for Applied Health Research and Delivery (WCAHRD), Division of Health Sciences, Warwick Medical School, University of Warwick, Coventry, United Kingdom; 3 Bloomberg School of Public Health, Johns Hopkins University, Baltimore, MD, United States of America; London School of Economics and Political Science, UNITED KINGDOM

## Abstract

**Background:**

Obstetric complications and maternal deaths can be prevented through safe delivery process. Facility based delivery significantly reduces maternal mortality by increasing women’s access to skilled personnel attendance. However, in sub-Saharan Africa, most deliveries take place without skilled attendants and outside health facilities. Utilization of facility-based delivery is affected by socio-cultural norms and several other factors including cost, long distance, accessibility and availability of quality services. This study examined country-level variations of the self-reported causes of not choosing to deliver at a health facility.

**Methods:**

Cross-sectional data on 37,086 community dwelling women aged between 15–49 years were collected from DHS surveys in Ethiopia (n = 13,053) and Nigeria (n = 24,033). Outcome variables were the self-reported causes of not delivering at health facilities which were regressed against selected sociodemographic and community level determinants. In total eight items complaints were identified for non-use of facility delivery: 1) Cost too much 2) Facility not open, 3) Too far/no transport, 4) don’t trust facility/poor service, 5) No female provider, 6) Husband/family didn’t allow, 7) Not necessary, 8) Not customary. Multivariable regression methods were used for measuring the associations.

**Results:**

In both countries a large proportion of the women mentioned facility delivery as not necessary, 54.9% (52.3–57.9) in Nigeria and 45.4% (42.0–47.5) in Ethiopia. Significant urban-rural variations were observed in the prevalence of the self-reported causes of non-utilisation. Women in the rural areas are more likely to report delivering at health facility as not customary/not necessary and healthy facility too far/no transport. However, urban women were more likely to complain that husband/family did not allow and that the costs were too high.

**Conclusion:**

Women in the rural were more likely to regard facility delivery as unnecessary and complain about transportation and financial difficulties. In order to achieving the maternal mortality related targets, addressing regional disparities in accessing maternal healthcare services should be regarded as a priority of health promotion programs in Nigeria and Ethiopia.

## Background

The period from 1990 to 2015 has witnessed a decline in maternal mortality ratio, albeit much slower than required to achieve the millennium development goals [1]. While displaying regional disparities, the levels of maternal mortality are “unacceptably high” in sub-Saharan Africa, about 66% of all maternal deaths worldwide. These “unacceptably high” maternal deaths are appalling, particularly when most of these maternal deaths can be prevented by utilizing the services of a skilled health personnel as in a health facility [2]. Several studies have highlighted the low utilization of maternal healthcare services, in particular health facility delivery in Nigeria [3,4,5] and Ethiopia [6,7,8]. However, there is a dearth of studies on subjectively reported underlying causes of poor utilization of health facility services in Nigeria. Accordingly, the current study aims to comprehend the underlying causes of low health facility utilization in Nigeria.

Skilled care throughout the period of pregnancy from inception to postnatal care is life saving for women and the child. There are proven medical strategies to prevent or treat, nearly 75% of complications of pregnancy including antepartum and postpartum haemorrhage, infections, puerperal complications, preeclampsia and eclampsia, and unsafe abortions, which can only be provided in a health facility set up [9,10]. Accordingly, the role of health facility delivery in preventing/treating pregnancy related complications and reducing maternal and neonatal mortality is indisputable. Despite this the utilization of health facilities and the rate of health facility delivery remains low in Nigeria with nearly one fifth of the deliveries occurring without anyone present during delivery [11,12]. Health facility delivery service utilization remains low in Ethiopia as well and as per one of the studies 85% of women in Ethiopia still deliver at home [13]

Research shows that universal health coverage entails broader demand side interventions toward maternal health access and health facility delivery is but one part of universal health access. The common strategies including provision of financial incentives, improving patient transfer and community involvement lack substantial quality evidence to corroborate their effectiveness and what is even more compelling is the lack of contextual appropriateness of such strategies [14]. On the other hand, although socioeconomic and cultural factors are highlighted, research studies have also focussed on supply side factors toward maternal healthcare services [15,16].

Without refuting the importance of studying multiple factors toward improving maternal healthcare service usage, it should be noted that results of many if not most of these studies are applicable to broader policy guidelines versus the need for locally applicable insights which may be dissimilar across regions even within the same country. In Nigeria, for example, midwives service scheme and The Subsidy Reinvestment and Empowerment Program (SURE-P) maternal and child health schemes were implemented with success to improve the maternal healthcare service usage. However, a dearth of active support for primary health centres by state and local governments has been noted and it is in this realm that studies such as the current one, are indispensable [17]. In fact, one research study has also noted uneven success of midwives’ service scheme in Nigeria with the challenges of varying levels of support from state and local governments [18]. In the same vein, studies from Ethiopia have reported the profound focus of research on individual and household factors affecting maternal healthcare service usage, while undermining the importance of contextual factors which in-fact play a crucial role in the success of the implemented programs [19,20].

In order to improve the support from these government actors and improve maternal health service utilization, local insights into the context of a given geopolitical zone is required. The study of regional disparities in the utilization of health facility delivery intra country and intercountry, particularly based on subjectively reported causes, aids providing such needed context[1,2]. The importance of regional barriers to accessing maternal healthcare was reported in low-income settings in South Asia e.g. Nepal. Nigeria and Ethiopia are two sub-Saharan African countries with similarities and differences, which further aid the study of and highlight the importance of contextual factors towards maternal healthcare service utilization, intra-country and intercountry. Comparing Nigeria and Ethiopia implies comparing two of the most populous African countries and also two countries in the list of 6, which account for more than 50% of maternal deaths globally [21]. Accordingly, this study aims to analyse the subjectively reported causes of non-usage of health facility delivery services within these two countries.

## Methods

### The survey and sampling design

We used cross-sectional data from recent Demographic and Health Surveys (DHSs) from Nigeria and Ethiopia conducted in 2013 and 2016 respectively. The DHSs were implemented in the respective countries with the financial and technical assistance by ICF International provisioned through the USAID-funded MEASURE DHS program. DHS surveys are nationally representative that collect information on a wide range of public health related topics such as anthropometric, demographic, socioeconomic, family planning and domestic violence to name a few. The survey covered men and women aged between 15–49 years and under-5 children residing in non-institutional settings (households excluding hospitals, cantonments). For sampling, a three-staged stratified cluster design was employed for Nigeria and two-stage design for Ethiopia. For Nigeria, the stages included selection of sampling strata, enumeration areas, and households, and for Ghana the stages include selection of enumeration areas and subsequent selections of households Cluster sampling serves as a time-efficient and cost-efficient technique to conduct population surveys in large geographical areas and select large samples. A more detailed version of the survey was published elsewhere [4,19].

### Variables selection and measurement

The dependent variables were self-reported reason of not delivering at health facilities. The following reasons were listed in the Nigeria DHS survey: 1. Not customary 2. Not necessary 3. Husband/family didn't allow 4. No female provider 5. Do not trust facility/poor service 6. Too far/no transport 7. Facility not open 8. Cost too much. The answers were coded as ‘Yes’ and ‘No’.

A set of potential confounding variables were included in the analysis as well based on their theoretical association with the outcome and explanatory variables in light of previous studies such as: Age of the participants (age groups): 15–19, 20–24, 25–29, 30–34, 35–39, 40–44, 45–49; Region: Residency: Urban, Rural; Religion: Christian, Islam, Other; Education: No education, Primary, Secondary; Employed: No, Yes; Wealth index: Poorest, Poorer, Middle, Richer, Richest; Sex of household head: Male, Female [1,4–9, 12,16–18].

For the calculation household wealth status, instead of direct income the volume of durable goods (e.g. TV, radio, bicycle) possessed by the household as well as and housing quality (e.g. type of floor, wall, and roof) are taken into consideration. Each item is assigned a factor score generated through principal component analysis (PCA) which are then summed and standardized for the households [22,23]. These standardised scores place the households in a continuous scale based on relative wealth scores. The scores thus obtained from a continuous scale and subsequently categorized into quintiles to rank the household as poorest/poorer/middle/richer/richest to richest.

### Data analysis

We did the analyses using publicly available data from demographic health surveys. Ethical procedures were the responsibility of the institutions that commissioned, funded, or managed the surveys. All DHS surveys are approved by ICF international as well as an Institutional Review Board (IRB) in respective country to ensure that the protocols are in compliance with the U.S. Department of Health and Human Services regulations for the protection of human subjects.

Since DHS surveys use complex sampling techniques, the usual analytical procedures are not suitable due the presence of cluster effects in the design. In order to adjust for this effect, the dataset was converted to a plan file accounting for the primary sampling units, sample strata and weight. This allowed complex sample analysis which is recommended for DHS data. After preparing the plan file, descriptive analyses were carried out to calculate the basic sociodemographic characteristics of the sample population and the reasons of not delivering at health facilities. These results were presented as percentages with 95% CIs. Differences in the percentages of the individual reasons between two countries were presented as bar charts. Following that, multivariable logistic regression analyses were performed to measure the odds ratios association between the individual causes and the main explanatory variables while adjusting for the confounders. The level of significance was set at p<0.05 for all analyses. All analyses were performed with SPSS version 24 for windows.

## Results

### Sample characteristics

Mean age of the women was 29.2 years (SD 7.15) and 29.1 years (SD 7.59) in Ethiopia and Nigeria respectively. Further sociodemographic profile was presented in Table 1. As the table indicates, majority of the women were in the age group of 25–29 years, of rural origin, followers of Christianity for Ethiopia and Islam for Nigeria, had no formal education, currently unemployed for Ethiopia and employed for Nigeria, were from the households of lowest wealth quintile, from male-headed households. In addition, the majority of women had 1–2 children in Ethiopia, but >4 children in Nigeria.

**Table 1:** Distribution of the explanatory variables.

	Ethiopia		Nigeria	
	n = 13,053	%	n = 24,033	%
**Age groups**				
15–19	794	6.1	1883	7.6
20–24	2637	20.2	4980	20.6
25–29	3648	27.9	6163	25.9
30–34	2527	19.4	4537	18.8
35–39	2052	15.7	3481	14.5
40–44	987	7.6	1998	8.2
45–49	408	3.1	991	4.3
Region				
**Residency**				
Urban	1617	12.4	4239	17.7
Rural	11436	87.6	19794	82.3
**Religion**				
Christian	7391	56.6	6705	27.2
Islam	5343	40.9	16725	70.1
Other	319	2.4	603	2.7
**Education**				
No education	7333	56.2	15703	65.4
Primary	3948	30.2	4796	19.9
Secondary	1163	8.9	3328	13.8
No education	609	4.7	15703	0.9
**Education of husband/partner**				
No education	7333	56.2	12671	52.7
Primary	3948	30.2	5337	22.2
Secondary	1163	8.9	4579	19.1
No education	609	4.7	1446	6.0
**Employed**				
No	9561	73.2	8942	36.0
Yes	3492	26.8	15091	64.0
**Wealth index**				
Poorest	3808	29.2	8372	35.0
Poorer	2508	19.2	7126	29.8
Middle	2368	18.1	4683	19.4
Richer	2206	16.9	2803	11.4
Richest	2163	16.6	1049	4.4
**Sex of household head**				
Male	10869	83.3	22034	91.5
Female	2184	16.7	1999	8.5
**Parity**				
1–2	7562	57.94	7049	29.33
3–4	2233	17.11	6452	26.85
>4	3258	24.95	10532	43.82

### Reasons for not delivering at health facilities

**Fig 1:**
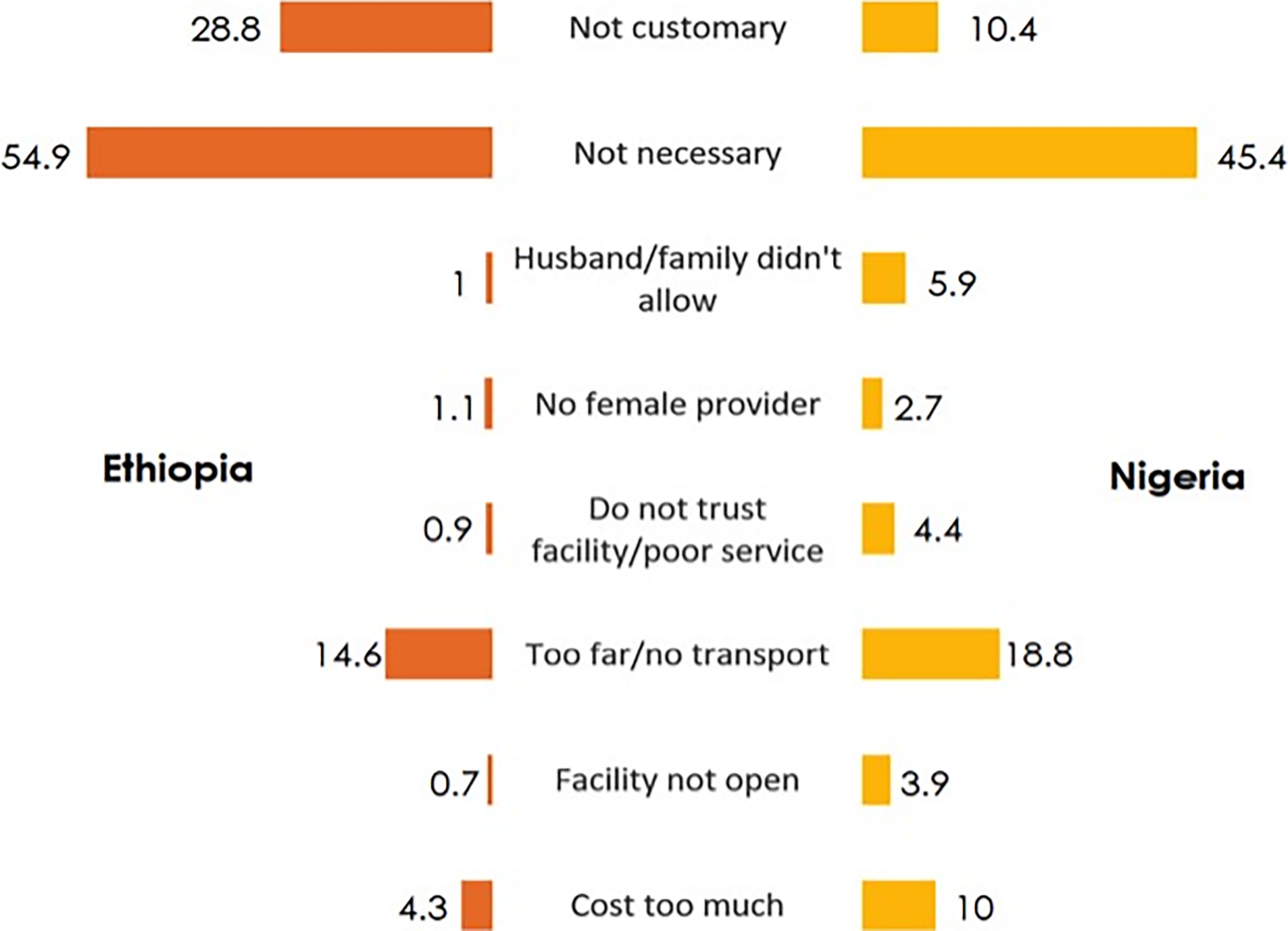
Comparison of the self-reported reasons for not delivering at health facilities in Ethiopia and Nigeria.

Fig 1 shows the percentages of the self-reported reasons for not delivering at a health facility. It is clear that majority of the women in both countries were not aware of the significance of health facility delivery as they described it as not necessary. More than a quarter of the women in Nigeria and one-tenth of in Ethiopia mentioned it as not customary. Apart from these, transportation and high expenses were reported by a significant proportion of the respondents.

### Urban-rural difference in the reasons for not delivering at health facilities

Self-reported reasons for not delivering at health facilities were presented separately for urban and rural women in Table 2. Women in the rural areas are more likely to report delivering at health facility as not customary (not a common practice in the family), not necessary (perceived unimportance of the service), health facility too far or no transport. However, urban women were more likely to complain that husband/family did not allow and that the costs were too high. Specifically, in the case of Ethiopia urban counterparts also reported issues related to absence of female provider, do not trust facility/poor service and health facility not open. This was however, not the case with Nigeria.

**Table 2:** Self-reported causes of not delivering at a health facility in Ethiopia and Nigeria.

	Ethiopia (%, 95% CI)		p	Nigeria (%, 95% CI)		p
	Urban	Rural		Urban	Rural	
**Not customary**	11.6 (7.4–17.8)	30.4, 28.6–32.3	0.001	7.6, 6.2–9.4	11.0, 9.7–12.5	0.013
**Not necessary**	48.0 (42.6–53.5	55.5, 53.6–57.4	0.003	42.7, 39.7–45.8	45.9, 44.3–47.5	0.343
**Husband/family didn't allow**	1.4, 0.6–3.7	1.0, 0.7–1.4	0.19	6.0, 4.9–7.2	5.8, 5.2–6.6	0.128
**No female provider**	1.3, 0.4–4.3	1.1, 0.6–2.0	0.215	0.8, 0.3–2.0	3.1, 2.2–4.4	0.001
**Do not trust facility/poor service**	1.6, 0.8–3.1	0.9, 0.5–1.5	0.37	4.4, 3.0–6.2	4.4, 3.4–5.7	0.39
**Too far/no transport**	5.2, 3.6–7.4	15.4, 14.0–17.0	<0.001	10.5, 8.8–12.6	20.4, 18.8–22.1	<0.001
**Facility not open**	1.1, 0.5–2.4	0.7, 0.5–0.9	0.146	2.1, 1.3–3.3	4.3, 3.6–5.1	0.007
**Cost too high**	4.7, 3.3–6.4	4.3, 3.7–4.9	0.209	11.1, 9.5–13.0	9.8, 9.0–10.7	0.115

Results of multivariable regression analysis were summarised in Table 3. It shows that urban women in Ethiopia had 75% significant reduction in the odds of health facility delivery as not customary, compared to rural women [OR = 0.248, 95%CI = 0.203–0.381], about 32% significant reduction in the odds of health facility delivery as not necessary, compared to the rural women [OR = 0.681, 95%CI = 0.409–0.918] and urban women were 1.465 times as likely to report health facility delivery is hindered by long distance or lack of transport, compared to rural women. In contrast, Nigerian urban women had 52% significant reduction in the odds of reporting health facility delivery is hindered by facility closed (not open), compared to rural women [OR = 0.475, 95%CI = 0.364–0.803] and cost been too much were 15% higher for urban women compared to the rural women [OR = 1.148, 95%CI = 1.015–1.298] (See Table 3 for details).

**Table 3:** Odds of the self-reported causes of not delivering at a health facility among urban women.

	Ethiopia		Nigeria	
	OR	95%CI	OR	95%CI
**Not customary **	0.248*	0.203–0.381	0.950	0.827–1.091
**Not necessary**	0.681*	0.409–0.918	0.981	0.777–1.239
**Husband/family didn't allow**	0.594	0.465–1.128	1.094	0.930–1.288
**No female provider**	0.636	0.386–2.031	0.774	0.470–1.519
**Do not trust facility/poor service**	1.221	0.803–1.858	0.764	0.636–1.31
**Too far/no transport**	1.465*	1.013–2.629	1.265	0.989–1.618
**Facility not open**	1.015	0.984–1.477	0.475*	0.364–0.803
**Cost too high**	1.083	0.742–2.115	1.179*	1.041–1.336

N.B. Reference category is rural residency.

OR = Odds ratio.

CI = Confidence interval.

* = significant at p<0.05.

Adjusted for age, residency, education, education of husband/partner, religion, employment, wealth index, household head, parity.

## Discussion

Health facility delivery “Not necessary”,”Not customary”, “Too far/no transport”, “Facility not open” and “Cost too high” were the chief factors reported in the study countries. As captured in the analysis, choosing not to delivering at health facility can be determined by tradition/perception of the problem, as well as structural and socioeconomic barrier. A surprisingly high number of women reported health facility delivery as unnecessary, indicative of the poor knowledge/awareness of the risks associated with childbirth without a skilled attendant. A greater proportion of the women who reported facility delivery as not customary and unnecessary had no educational qualification (not shown in the analysis). Women with higher educational level, in both urban and rural areas had lower likelihood of regarding facility delivery as unnecessary/not customary, and higher likelihood of complaining about the opening hours of health facility. The percentages of complaining about cost and distance to health facility did not differ significantly among the categories of education.

The results of our analyses showed that ‘cost’ is one of the top four barriers reported for not attending health facility delivery in both urban and rural areas, with slightly higher likelihood of such reports in urban areas. This remains true for both the countries, Nigeria and Ethiopia. It is in line with previous research studies where ‘cost’ remains an influential factor in determining the place of delivery. One study, for example reported cost of delivery as prohibitive particularly for those with seasonal work [24]. Another study reported cost as a barrier although not for all the women and the consideration of traditional birth attendants as an alternative to health facility delivery due to cost [25,26]. Cost has been reported as a barrier to maternal healthcare service utilization in the studies for Ethiopia and Nigeria [27,28]. These usually have a policy implication in terms of removing user fees or in some way providing free/subsidized access to maternal healthcare services, which although not inappropriate but may be insufficient on its own [28]. These policy measures for example, in many cases disregard the indirect costs associated with health facility delivery/maternal healthcare service utilization as well as other barriers as discussed below [28]. Thus, cost remains one of the many factors which impede health facility delivery service utilization.

However, about one in four women in Ethiopia and one in ten women Nigeria reported that “Not necessary/Not customary” as reasons for not attending health facility delivery. Although the current study lacked data to infer on the precise reasons underlying greater reporting of such factors, particularly in Ethiopia, there was a related study which highlighted the concept of considering delivery as a natural phenomenon versus a health ailment requiring health facility services in Ethiopia. This study also elaborated on the pleasure, women receive from rituals following home delivery [29]. On the other hand in the Nigerian context lack of education has been cited as the underlying factor behind the “Not necessary/Not customary” reports of health facility delivery and further that planning health facility delivery in advance reduces such reports [30,31] Lack of awareness/misconception of the importance of health facility delivery and socio-cultural factors have been cited in previous research studies consistent with the findings of the current study [32,33,34].

In the same vein, transportation still remains an issue determining health facility delivery. Transportation issue is more likely to be reported in urban areas for Ethiopian women. One study for example reported transportation issues more so in the rural context and the issue of poverty limiting alternative transportation provision [35]. Another study identified ‘access’ barriers to health facility delivery and the need for tools to identify regional disparities in ‘access’ and further utilizing these tools toward healthcare planning by local officials [36]. Studies such as the current one become important in this context since it not only identifies barriers (subjectively reported) but also aims to highlight the regional areas needing improvement. Although it is true for both the countries, Ethiopia has higher prevalence of reports of transportation issues and accordingly might have greater implications for Ethiopia than Nigeria.

A few women also reported issues of trust for the provider/poor service as a factor in determining health facility delivery. To corroborate these findings, one previous study reported poor quality of services as detrimental to health facility deliveries, particularly in the realm of ‘process’ i.e. standards of care employed in the provision of these services [37,38]. However, in the current study we were limited by the data availability and accordingly not in a position to comment on which indicators of quality were compromised. Absence of female providers although not a major reason (even in the current study) has been cited in one previous study from Bangladesh [39]. Interestingly some women reported the issue of husband/family not allowing health facility delivery. The issue of husband/family not allowing health facility delivery has been identified by one other research study [40].

Again, women in rural areas were consistently more likely to report most of the issues above as reasons for not delivery at health facility relative to their urban counterparts. Facility not being open, transportation issues, absence of female provider and that health facility delivery was not necessary/not customary were more likely to be reported in the rural areas relative their urban counterparts. These findings are consistent with previous research studies [41,42,43].

### Strengths and limitations

The results of the study can be used by local policy makers to target the reasons in a given area for improving maternal healthcare services and their usage. However, the current is limited by the availability of data and accordingly certain aspects could not be elaborately analysed. For example, ‘cost’ has been noted as one barrier to delivering in health facility. But it could not be analysed that this cost is due to loss of wages or any other aspect related to the use of maternal healthcare services. Similarly, when it comes to poor services it is not clear whether the attitude of the healthcare staff or process related issues in services were below par standards. In the same vein when family not approving health facility delivery was a reason it was not possible to elaborate on the reasons as to why the husband/family were not approving the same. Also, potential confounders like parity, marital status and antenatal care uptake were not included in the analysis owing to the limited scope of the project. The surveys in Ethiopia and Nigeria were conducted three years apart, which might account for large variation in the self-reported causes of not delivering at a health facility. Another important limitation is the use of preselected list of questions on not delivering a health facility, a construct which is essentially qualitative in nature. Future studies should focus on exploring the root causes especially sociocultural ones through qualitative in-depth interviews and not close-ended questions as used in the present study.

## Conclusions

Utilization of health facility delivery is impacted by multiple factors in both the countries, Nigeria and Ethiopia. Major reports included health facility delivery as “Not necessary/Not customary”, “Cost too high” and “Too far/no transport”. Specific policy implications might include considering reduction/elimination of costs associated with delivery, both direct and indirect, through schemes like user fee elimination and/or subsidized/free maternal healthcare services. This might also make a case for universal health coverage. However, such schemes should be cautious in expecting the outcome of the schemes since there are multiple other factors playing a role. For example, transportation initiatives might be considered focussing on the rural set up for both the countries. Also, in the context of Ethiopia a focus on increasing the awareness of the need for health facility delivery through public health marketing may be one option. In the same vein, for Nigeria a focus on improving education and improving antenatal coverage to establish planned deliveries, can be considered. In order to achieving the maternal mortality related targets, addressing regional disparities in accessing maternal healthcare services should be regarded as a priority of health promotion programs in Nigeria and Ethiopia. Local policy makers should take into account these findings while framing appropriate policies toward providing maternal healthcare services and improve their usage accordingly. There is also need for more studies highlighting the regional disparities in maternal healthcare service usage.

## References

[1] Alkema L, Chou D, Hogan D, Zhang S, Moller A-B, Gemmill A, et al Global, regional, and national levels and trends in maternal mortality between 1990 and 2015, with scenario-based projections to 2030: a systematic analysis by the UN Maternal Mortality Estimation Inter-Agency Group. The Lancet. 2016;387(10017):462–74. 10.1016/S0140-6736(15)00838-7 PMC5515236 26584737

[2] Maternal Mortality [Internet]. UNICEF DATA. [cited 2017Nov30]. Available from: https://data.unicef.org/topic/maternal-health/maternal-mortality/

[3] Moore BM, Alex-Hart BA, George IO. Utilization of health care services by pregnant mothers during delivery: a community based study in Nigeria. East African Journal of Public Health. 2011 3;8(1):49–51. 22066284

[4] Austin A, Fapohunda B, Langer A, Orobaton N. Trends in delivery with no one present in Nigeria between 2003 and 2013. International Journal of Womens Health. 2015;:345. 10.2147/IJWH.S79573 PMC4396652 25897265

[5] Ononokpono DN, Odimegwu CO. Determinants of Maternal Health Care Utilization in Nigeria: a multilevel approach. Pan African Medical Journal. 2014;17. 10.11694/pamj.supp.2014.17.1.3596 PMC3958146 24643545

[6] Maternal mortality [Internet]. World Health Organization. World Health Organization; [cited 2017Dec15]. Available from: http://www.who.int/mediacentre/factsheets/fs348/en/

[7] Shigute T, Tejineh S, Tadesse L. Institutional Delivery Service Utilization and Associated Factors among Women of Child Bearing Age at Boset Woreda, Oromia Regional State, Central Ethiopia. Journal of Womens Health Care. 2017;06(05).

[8] Hamdela B. Predictors of Health Facility Delivery Service Utilization in Lemo District, South Ethiopia: Unmatched Case Control Study. Journal of Pregnancy and Child Health. 2015;02(02).

[9] Bhattacharyya S, Srivastava A, Roy R, Avan BI. Factors influencing women’s preference for health facility deliveries in Jharkhand state, India: a cross sectional analysis. BMC Pregnancy and Childbirth. 2016 7;16(1). 10.1186/s12884-016-0839-6 PMC4782569 26951787

[10] Kidanu S, Degu G, Tiruye TY. Factors influencing institutional delivery service utilization in Dembecha district, Northwest Ethiopia: A community based cross sectional study. Reproductive Health. 2017;14(1). 10.1186/s12978-017-0359-5 PMC5567650 28830523

[11] Fapohunda BM, Orobaton NG. When Women Deliver with No One Present in Nigeria: Who, What, Where and So What? PLoS ONE. 2013;8(7). 10.1371/journal.pone.0069569 PMC3723888 23936047

[12] Adebayo AM, Asuzu MC. Utilisation of a community-based health facility in a low-income urban community in Ibadan, Nigeria. African Journal of Primary Health Care & Family Medicine. 2015 11;7(1). 10.4102/phcfm.v7i1.735 PMC4564905 26245600

[13] Demilew YM, Gebregergs GB, Negusie AA. Factors associated with institutional delivery in Dangila district, North West Ethiopia: a cross-sectional study. African Health Sciences. 2016 9;16(1):10 doi: 10.4314/ahs.v16i1.2 27358608 10.4314/ahs.v16i1.2 PMC4915427

[14] Elmusharaf K, Byrne E, O’Donovan D. Strategies to increase demand for maternal health services in resource-limited settings: challenges to be addressed. BMC Public Health. 2015 8;15(1). doi: 10.1186/s12889-015-2459-x 10.1186/s12889-015-2222-3 PMC4562346 26350731

[15] Gage AJ, Ilombu O, Akinyemi AI. Service readiness, health facility management practices, and delivery care utilization in five states of Nigeria: a cross-sectional analysis. BMC Pregnancy and Childbirth. 2016 6;16(1). 10.1186/s12884-016-1097-3 PMC5054586 27716208

[16] Singh A. Supply-side barriers to maternal health care utilization at health sub-centers in India. PeerJ. 2016 3;4. 10.7717/peerj.2675 PMC5101621 27833824

[17] Five Ways an Innovative Program Increased Facility Birth in Nigeria [Internet]. Maternal Health Task Force. 2016 [cited 2017Dec21]. Available from: https://www.mhtf.org/2015/01/13/five-ways-an-innovative-program-increased-facility-birth-in-nigeria/

[18] Abimbola S, Okoli U, Olubajo O, Abdullahi MJ, Pate MA. The Midwives Service Scheme in Nigeria. PLoS Medicine. 2012 1;9(5). 10.1371/journal.pmed.1001211 PMC3341343 22563303

[19] Mezmur M, Navaneetham K, Letamo G, Bariagaber H. Individual, household and contextual factors associated with skilled delivery care in Ethiopia: Evidence from Ethiopian demographic and health surveys. Plos One. 2017;12(9). 10.1371/journal.pone.0184688 PMC5598994 28910341

[20] Lassi ZS, Kumar R, Bhutta ZA. Community-Based Care to Improve Maternal, Newborn, and Child Health. Disease Control Priorities, Third Edition (Volume 2): Reproductive, Maternal, Newborn, and Child Health. 2016Nov;:263–84.

[21] Berhan Y, Berhan A. Review of Maternal Mortality in Ethiopia: A Story of the Past 30 Years. Ethiopian Journal of Health Sciences. 2014 12;24:3. 10.4314/ejhs.v24i0.2s PMC4249207 25489179

[22] Vyas S., Kumaranayake L. Constructing socio-economic status indices: how to use principal components analysis. Health Policy Plan. 2006;21(6): 459–68. doi: 10.1093/heapol/czl029 17030551 10.1093/heapol/czl029

[23] Montgomery M.R et al Measuring living standards with proxy variables. Demography. 2000; 37(2):155–74. 10836174

[24] Bohren MA, Hunter EC, Munthe-Kaas HM, Souza JP, Vogel JP, Gülmezoglu AM. Facilitators and barriers to facility-based delivery in low- and middle-income countries: a qualitative evidence synthesis. Reproductive Health. 2014;11(1). 10.1186/1742-4755-11-71 PMC4247708 25238684

[25] Mason L, Dellicour S, Kuile FT, Ouma P, Phillips-Howard P, Were F, et al Barriers and facilitators to antenatal and delivery care in western Kenya: a qualitative study. BMC Pregnancy and Childbirth. 2015;15(1). 10.1186/s12884-015-0453-z PMC4358726 25886593

[26] Montagu D, Yamey G, Visconti A, Harding A, Yoong J. Where Do Poor Women in Developing Countries Give Birth? A Multi-Country Analysis of Demographic and Health Survey Data. PLoS ONE. 2011;6(2). 10.1371/journal.pone.0017155 PMC3046115 21386886

[27] Sim KBBR Dhakal DKS. Analyzing Barriers to Accessing Health Care Services in Holeta Town, Ethiopia. Primary Health Care Open Access. 2015;05(02).

[28] Edu B, Agan TU, Monjok E, Makoweicka K. Effect of Free Maternal Health Care Program on Health-seeking Behavior of Women during Pregnancy, Intra-partum and Postpartum Periods in Cross River State of Nigeria: A Mixed Method Study. Open Access Macedonian Journal of Medical Sciences. 2017; 10.3889/oamjms.2017.075 PMC5503739 28698759

[29] Kaba M, Bulto T, Tafesse Z, Lingerih W, Ali I. Sociocultural determinants of home delivery in Ethiopia: a qualitative study. International Journal of Womens Health. 2016;:93. 10.2147/IJWH.S98722 PMC4833377 27114718

[30] Adewuyi EO, Zhao Y, Auta A, Lamichhane R. Prevalence and factors associated with non-utilization of healthcare facility for childbirth in rural and urban Nigeria: Analysis of a national population-based survey. Scandinavian Journal of Public Health. 2017;45(6):675–82. doi: 10.1177/1403494817705562 28653565 10.1177/1403494817705562

[31] Mph JEM, Pharr PJR, Wyk PBV, Mph EEEM. Factors Influencing the Choice of Child Delivery Location among Women Attending Antenatal Care Services and Immunization Clinic in Southeastern Nigeria. International Journal of MCH and AIDS (IJMA). 2017;6(1):82. 28798897 10.21106/ijma.213 PMC5547229

[32] Roro M, Hassen E, Lemma A, Gebreyesus S, Afework M. Why do women not deliver in health facilities: a qualitative study of the community perspectives in south central Ethiopia? BMC Research Notes. 2014;7(1):556. 25143017 10.1186/1756-0500-7-556 PMC4155096

[33] Kitui J, Lewis S, Davey G. Factors influencing place of delivery for women in Kenya: an analysis of the Kenya demographic and health survey, 2008/2009. BMC Pregnancy and Childbirth. 2013;13(1). 10.1186/1471-2393-13-40 PMC3585789 23414104

[34] Kumbani L, Bjune G, Chirwa E, Malata A, Odland JØ. Why some women fail to give birth at health facilities: a qualitative study of women’s perceptions of perinatal care from rural Southern Malawi. Reproductive Health. 2013 8;10(1). 10.1186/1742-4755-10-9 PMC3585850 23394229

[35] Atuoye KN, Dixon J, Rishworth A, Galaa SZ, Boamah SA, Luginaah I. Can she make it? Transportation barriers to accessing maternal and child health care services in rural Ghana. BMC Health Services Research. 2015;15(1). 10.1186/s12913-015-1005-y PMC4545969 26290436

[36] Parkhurst JO, Ssengooba F. Assessing access barriers to maternal health care: measuring bypassing to identify health centre needs in rural Uganda. Health Policy and Planning. 2009 11;24(5):377–84. doi: 10.1093/heapol/czp023 19520736 10.1093/heapol/czp023

[37] Fisseha G, Berhane Y, Worku A, Terefe W. Quality of the delivery services in health facilities in Northern Ethiopia. BMC Health Services Research. 2017 9;17(1). 10.1186/s12913-017-2125-3 PMC5345168 28279215

[38] Hernandez B, Colombara DV, Gagnier MC, Desai SS, Haakenstad A, Johanns C, et al Barriers and facilitators for institutional delivery among poor Mesoamerican women: a cross-sectional study. Health Policy and Planning. 2017;32(6):769–80. doi: 10.1093/heapol/czx010 28335004 10.1093/heapol/czx010

[39] Sarker BK, Rahman M, Rahman T, Hossain J, Reichenbach L, Mitra DK. Reasons for Preference of Home Delivery with Traditional Birth Attendants (TBAs) in Rural Bangladesh: A Qualitative Exploration. Plos One. 2016 5;11(1). 10.1371/journal.pone.0146161 PMC4701391 26731276

[40] Onta S, Choulagai B, Shrestha B, Subedi N, Bhandari GP, Krettek A. Perceptions of users and providers on barriers to utilizing skilled birth care in mid- and far-western Nepal: a qualitative study. Global Health Action. 2014 11;7(1):24580. 25119066 10.3402/gha.v7.24580 PMC4131000

[41] Dickson KS, Adde KS, Amu H. What Influences Where They Give Birth? Determinants of Place of Delivery among Women in Rural Ghana. International Journal of Reproductive Medicine. 2016;2016:1–8. 10.1155/2016/7203980 PMC5215620 28101522

[42] Stock R. Distance and the utilization of health facilities in rural Nigeria. Social Science & Medicine. 1983;17(9):563–70. 6879255 10.1016/0277-9536(83)90298-8

[43] Nwakoby BN. The influence of new maternal care facilities in rural Nigeria. Health Policy and Planning. 1992;7(3):269–78.

